# Real world, big data cost of pharmaceutical treatment for rheumatoid arthritis in Greece

**DOI:** 10.1371/journal.pone.0226287

**Published:** 2019-12-12

**Authors:** Kyriakos Souliotis, Christina Golna, Chara Kani, Sofia Nikolaidi, Dimitrios Boumpas

**Affiliations:** 1 Faculty of Social and Political Sciences, University of Peloponnese, Corinth, Greece; 2 Health Policy Institute, Athens, Greece; 3 Innowth Ltd, Larnaca, Cyprus; 4 Medicines Division, National Organization for Healthcare Services Provision (EOPYY), Athens, Greece; 5 Attikon University Hospital, National and Kapodistrian University of Athens, Athens, Greece; Universita Campus Bio-Medico di Roma, ITALY

## Abstract

**Introduction:**

Rheumatoid Arthritis (RA) is a highly prevalent autoimmune disease associated with joint inflammation and destruction. Treatment for RA, especially with biologic agents (biologics), improves patient functionality and quality of life and averts costly complications or disease progression. Cost of RA pharmaceutical treatment has rarely been reported on the basis of real-world, big data. This study reports on the real-world, big data RA pharmaceutical treatment cost in Greece.

**Methods:**

The Business Intelligence database of the National Organization for Healthcare Services Provision (EOPYY) was used to identify and provide analytics on patients on treatment for RA. EOPYY is responsible for funding healthcare and pharmaceutical care services for approximately 95% of the population in the country. ICD-10 codes were applied to identify patients with RA and at least one reimbursed prescription between 1 June 2014 and 31 May 2015.

**Results:**

35,873 unique patients were recorded as undergoing treatment for RA. Total reimbursed treatment cost for the study period was €81,206,363.70, of which €52,732,142.18 (64.94%) was for treatment with biologics. Of that cost, €39,724,489.71 (48.32%) accounted for treatment with anti-TNFs and/or methotrexate/corticosteroids.

**Conclusion:**

Real world, big data analysis confirms that the major driver of RA pharmaceutical cost is, as expected, the cost of treatment with biologics. It is critical to be able to match this cost to the treatment outcome it produces to ensure an optimal, no-waste, evidence-based allocation of healthcare resources to need.

## Introduction

Rheumatoid arthritis (RA) is the most common type of chronic autoimmune disorder that primarily affects joints [1]. Its prevalence is estimated at approximately 1% worldwide [2] and between 0.68% [3] and 0.84% [4] in Greece, being more common among women than men. RA carries a substantial morbidity burden, which impacts on patient quality of life [5], as well as a significant financial burden, as it reduces patient capacity to work [6] and increases direct and indirect healthcare costs [7,8], for the patients and their families, the health care system and the society as a whole.

In order to relieve pain and avoid irreversible joint destruction and disability, RA requires early, goal-oriented treatment with timely adjustment. Drugs used for the treatment of RA are non-steroid anti-inflammatory drugs that have rapid onset of action but do not alter the course of disease, corticosteroids that suppress synovitis and the symptoms of RA, disease modifying anti-rheumatic drugs (DMARDs) and biological agents (biologics), including anti–TNF agents (anti-TNFs) alone or in combination with other options. Biologics are more expensive than other treatment options and, therefore, usually reserved for subsequent treatment lines, once other options have been exhausted. The effectiveness and cost-effectiveness of biologic agents for the treatment of RA has been thoroughly investigated and, in most cases, well documented [9].

In Greece, physicians are relatively free to select the clinically appropriate treatment option for their RA patient, though different options are reimbursed differently by the National Organization for Healthcare Services (EOPYY), which is responsible for funding health care and pharmaceutical care services for 95% of the population in the country. As detailed in previous studies for related autoimmune conditions [10], treatment with a biologic agent is reimbursed at 100% of the cost, whereas treatment with non-biologics carries a 25% copayment fee for the patient, to which any difference in price between the product dispensed and the lowest priced generic alternative is added.

As EOPYY is looking to introduce disease related global budgets to better manage treatment provision and allow for substantial economies, it is critical to understand the actual, real world burden of such conditions, in terms of both health and costs, if to ensure a budget is set that caters for actual patient need, leaving no one behind. This study is the first analysis and publication of actual, real world, big data pharmaceutical expenditure for the treatment of RA in Greece.

## Materials and methods

This is a retrospective, observational study based on EOPYY’s anonymized health administrative data for the period between June 2014 and May 2015. The Business Intelligence database of EOPYY was used to provide analytics on individuals (date of birth and gender), based on the unique citizens’ social security number (AMKA). Eligibility criteria included unique patients, who had received at least one reimbursed pharmacotherapy through the e-prescription system for predefined ICD-10 codes (M05, M05.0, M05.1, M05.2, M05.3, M05.8, M05.9, M06, M06.0, M06.4, M06.8, M06.9). As reported elsewhere [10], the study period was determined to maximize population coverage and quality of data, since almost 95% of the Greek population was registered in the EOPYY database by June 2014. To avoid double counting, each unique patient was matched to the most frequently reported predefined RA ICD 10 code for the period under study.

Permission to use anonymized data was obtained by the administration of EOPYY (approval decision of the President / protocol number C99/2317/1.10.2015), in accordance with the national legislation on the Protection of Individuals with regards to the Processing of Personal Data. The study has been approved by the Research Ethics Committee of the University of Peloponnese.

Patient demographics (age and gender), type and number of treatments administered for RA (DMARDs, anti-TNF agents, corticosteroids, methotrexate, other biologics) and cost per therapy option were retrieved from the database. Total and average per unique patient annual pharmaceutical cost to EOPYY per unique patient was calculated per pharmacotherapy option.

This analysis excludes sales of pharmaceuticals purchased out of pocket by patients. Cost of pharmaceuticals was calculated at list price, without deducting additional rebates and discounts to EOPYY. Efficacy and safety were not analyzed and can be considered similar to those reported in a network meta-analysis on biological agents [11].

## Results

A total number of 35,873 unique patients were recorded as undergoing pharmaceutical treatment for RA during the study period. The vast majority were female (78.7%) and over 65 years old (57.9%). Table 1 depicts patient age and gender distribution.

**Table 1:** Age group distribution of RA patients by sex.

	Females	% Females	Males	% Males	Total	% Total
**5–14**	20	0.1%	6	0.1%	26	0.1%
**15–24**	169	0.6%	74	1.0%	243	0.7%
**25–34**	557	2.0%	142	1.9%	699	1.9%
**35–44**	1549	5.5%	389	5.1%	1938	5.4%
**45–54**	3581	12.7%	818	10.7%	4399	12.3%
**56–64**	6355	22.5%	1440	18.9%	7795	21.7%
**65–74**	7315	25.9%	2011	26.4%	9326	26.0%
**75-..**	8704	30.8%	2748	36.0%	11452	31.9%
**Total**	28250	100.0%	7628	100.0%	35878	100.0%

Table 2 presents distribution of patients by pharmacotherapy option. 12,275 patients (34.2%) were on a corticosteroid and/or methotrexate, 3,535 (9.9%) and 2,647 (7.4%) of whom on corticosteroids and methotrexate as monotherapy, respectively. 4,952 patients (13.8%) were on treatment with anti-TNFs and/or methotrexate and/or corticosteroids, of whom only 3.7% on anti-TNFs as monotherapy. Almost 5% of patients were on treatment with other biologics with or without corticosteroids or methotrexate and 12.6% were on DMARDs as monotherapy. More than a third of the patients (12,363–34.5%) were on treatment with various combinations of the abovementioned treatment options.

**Table 2:** Distribution of patients by pharmacotherapy option.

Type of treatment (monotherapies and combinations)	Unique Patients (N)	% of total
DMARDs	4531	12.6%
	4531	12.6%
CS	3535	9.9%
CS + MTX	6093	17.0%
MTX	2647	7.4%
	12275	34.2%
ANTI-TNFs	1341	3.7%
ANTI-TNFs + CS	683	1.9%
ANTI-TNFs + CS + MTX	1651	4.6%
ANTI-TNFs + MTX	1277	3.6%
	4952	13.8%
OTHER BIOLOGICS	467	1.3%
OTHER BIOLOGICS + CS	372	1.0%
OTHER BIOLOGICS + CS + MTX	598	1.7%
OTHER BIOLOGICS + MTX	315	0.9%
	1752	4.9%
ANTI-TNFs + CS + DMARDs	813	2.3%
ANTI-TNFs + CS + DMARDs + MTX	471	1.3%
ANTI-TNFs + CS + DMARDs + MTX + OTHER BIOLOGICS	52	0.1%
ANTI-TNFs + CS + DMARDs + OTHER BIOLOGICS	51	0.1%
ANTI-TNFs + CS + MTX + OTHER BIOLOGICS	111	0.3%
ANTI-TNFs + CS + OTHER BIOLOGICS	42	0.1%
ANTI-TNFs + DMARDs	497	1.4%
ANTI-TNFs + DMARDs + MTX	144	0.4%
ANTI-TNFs + DMARDs + MTX + OTHER BIOLOGICS	8	0.0%
ANTI-TNFs + DMARDs + OTHER BIOLOGICS	9	0.0%
ANTI-TNFs + MTX + OTHER BIOLOGICS	31	0.1%
ANTI-TNFs + OTHER BIOLOGICS	30	0.1%
CS + DMARDs	5579	15.5%
CS + DMARDs + MTX	2524	7.0%
CS + DMARDs + MTX + OTHER BIOLOGICS	229	0.6%
CS + DMARDs + OTHER BIOLOGICS	409	1.1%
DMARDs + MTX	1140	3.2%
DMARDs + MTX + OTHER BIOLOGICS	50	0.1%
DMARDs + OTHER BIOLOGICS	173	0.5%
	12363	34.5%
Total	35873	100.0%

Note: CS = Corticosteroids, MTX = Methotrexate

Table 3 presents overall patient age distribution per therapeutic combination. The majority of patients treated with corticosteroids and/or methotrexate were over 75 years old (42.1%), followed by those aged 65–74 (25.1%). Similarly, more than 50% (56.7%) of patients treated with DMARDs as monotherapy were over 65 years old. Other biologics were primarily prescribed to middle-aged patients (aged 56–64), closely followed by those aged 65–74 (28.3% and 28% respectively). Within age groups, the majority of patients under 34 were treated with anti-TNFs (with or without methotrexate and/or corticosteroids) and over 35 with corticosteroids, with or without methotrexate.

**Table 3:** Patient distribution per pharmacotherapy option and age group.

	5–14	15–24	25–34	35–44	45–54	55–64	65–74	75-..	Total
**DMARDs**	1	23	66	208	600	1068	1176	1389	4531
	1	23	66	208	600	1068	1176	1389	4531
**CS**	2	5	18	72	169	347	617	2305	3535
**CS + MTX**	4	34	101	291	653	1190	1687	2133	6093
**MTX**	8	22	38	126	331	619	779	724	2647
	14	61	157	489	1153	2156	3083	5162	12275
**ANTI-TNFs**	-	28	101	165	269	343	253	182	1341
**ANTI-TNFs + CS**	-	2	18	48	77	148	177	213	683
**ANTI-TNFs + CS + MTX**	2	14	34	102	249	442	492	316	1651
**ANTI-TNFs + MTX**	8	34	34	94	204	375	354	174	1277
	10	78	187	409	799	1308	1276	885	4952
**OTHER BIOLOGICS**	-	7	21	53	68	126	111	81	467
**OTHER BIOLOGICS + CS**	-	1	6	13	36	98	112	106	372
**OTHER BIOLOGICS + CS + MTX**	-	3	14	28	80	175	185	113	598
**OTHER BIOLOGICS + MTX**	-	8	14	21	46	96	82	48	315
	0	19	55	115	230	495	490	348	1752
**ANTI-TNFs + CS + DMARDs**	-	6	21	68	129	208	219	162	813
**ANTI-TNFs + CS + DMARDs + MTX**	-	2	18	48	96	130	106	71	471
**ANTI-TNFs + CS + DMARDs + MTX + OTHER BIOLOGICS**	-	3	-	6	14	13	10	6	52
**ANTI-TNFs + CS + DMARDs + OTHER BIOLOGICS**	-	1	1	4	12	13	9	11	51
**ANTI-TNFs + CS + MTX + OTHER BIOLOGICS**	-	-	2	10	18	31	33	17	111
**ANTI-TNFs + CS + OTHER BIOLOGICS**	-	-	1	2	3	9	15	12	42
**ANTI-TNFs + DMARDs**	-	7	13	44	90	127	133	83	497
**ANTI-TNFs + DMARDs + MTX**	-	3	9	15	34	42	32	9	144
**ANTI-TNFs + DMARDs + MTX + OTHER BIOLOGICS**	-	-	-	-	1	6	1	-	8
**ANTI-TNFs + DMARDs + OTHER BIOLOGICS**	-	-	-	1	1	1	5	1	9
**ANTI-TNFs + MTX + OTHER BIOLOGICS**	-	-	-	3	3	13	6	6	31
**ANTI-TNFs + OTHER BIOLOGICS**	-	1	1	1	6	7	8	6	30
**CS + DMARDs**	-	11	82	247	511	1034	1443	2251	5579
**CS + DMARDs + MTX**	-	17	47	154	381	611	670	644	2524
**CS + DMARDs + MTX + OTHER BIOLOGICS**	-	-	5	24	38	73	50	39	229
**CS + DMARDs + OTHER BIOLOGICS**	-	2	12	14	54	95	137	95	409
**DMARDs + MTX**	1	8	16	62	191	289	351	222	1140
**DMARDs + MTX + OTHER BIOLOGICS**	-	-	1	2	9	20	15	3	50
**DMARDs + OTHER BIOLOGICS**	-	-	5	12	26	44	57	29	173
	1	61	234	717	1617	2766	3300	3667	12363
**Total**	26	243	699	1938	4399	7795	9326	11452	35878

Note: CS = Corticosteroids, MTX = Methotrexate

Total annual cost for reimbursed pharmaceuticals for the treatment of RA during the study year was calculated at €81,206,363.70. Biologics accounted for almost 70% of total spent (€52,732,142.18–64.94%). More specifically, treatment with anti-TNFs with or without corticosteroids/methotrexate accounted for almost 50% of total spent (48.92%, €39,724,489.71) and treatment with other biologics (with or without corticosteroids/methotrexate) accounted for 16.02% (€13,007,652.47). Treatment with anti-TNFs as monotherapy had a mean annual per patient expenditure of €7,681. This rose to €8,488.19, when anti-TNFs were combined with methotrexate. Table 4 presents total and average expenditure per pharmacotherapy option for the study year.

**Table 4:** Pharmacotherapy costs for RA, June 2014- June 2015.

	Unique Patients (N)	Average annual cost per patient	Expenditure	% of Total
DMARDs	4531	154.14 €	698,404.77 €	0.86%
	4531	154.14 €	698,404.77 €	0.86%
CS	3535	24.98 €	88,296.18 €	0.11%
CS + MTX	6093	149.35 €	909,966.16 €	1.12%
MTX	2647	129.73 €	343,400.55 €	0.42%
	12275	109.30 €	1,341,662.89 €	1.65%
ANTI-TNFs	1341	7,681.85 €	10,301,364.98 €	12.69%
ANTI-TNFs + CS	683	7,766.09 €	5,304,239.59 €	6.53%
ANTI-TNFs + CS + MTX	1651	8,043.28 €	13,279,462.30 €	16.35%
ANTI-TNFs + MTX	1277	8,488.19 €	10,839,422.85 €	13.35%
	4952	8,021.91 €	39,724,489.71 €	48.92%
OTHER BIOLOGICS	467	7,062.66 €	3,298,262.96 €	4.06%
OTHER BIOLOGICS + CS	372	7,334.20 €	2,728,322.79 €	3.36%
OTHER BIOLOGICS + CS + MTX	598	7,441.87 €	4,450,236.35 €	5.48%
OTHER BIOLOGICS + MTX	315	8,034.38 €	2,530,830.36 €	3.12%
	1752	7,424.46 €	13,007,652.47 €	16.02%
ANTI-TNFs + CS + DMARDs	813	7,824.53 €	6,361,343.02 €	7.83%
ANTI-TNFs + CS + DMARDs + MTX	471	7,000.85 €	3,297,400.61 €	4.06%
ANTI-TNFs + CS + DMARDs + MTX + OTHER BIOLOGICS	52	9,229.34 €	479,925.78 €	0.59%
ANTI-TNFs + CS + DMARDs + OTHER BIOLOGICS	51	9,713.19 €	495,372.63 €	0.61%
ANTI-TNFs + CS + MTX + OTHER BIOLOGICS	111	9,993.56 €	1,109,285.47 €	1.37%
ANTI-TNFs + CS + OTHER BIOLOGICS	42	8,532.37 €	358,359.73 €	0.44%
ANTI-TNFs + DMARDs	497	8,067.08 €	4,009,339.41 €	4.94%
ANTI-TNFs + DMARDs + MTX	144	8,228.54 €	1,184,909.72 €	1.46%
ANTI-TNFs + DMARDs + MTX + OTHER BIOLOGICS	8	11,098.15 €	88,785.21 €	0.11%
ANTI-TNFs + DMARDs + OTHER BIOLOGICS	9	9,162.85 €	82,465.63 €	0.10%
ANTI-TNFs + MTX + OTHER BIOLOGICS	31	9,562.58 €	296,439.97 €	0.37%
ANTI-TNFs + OTHER BIOLOGICS	30	10,474.07 €	314,222.04 €	0.39%
CS + DMARDs	5579	192.97 €	1,076,578.15 €	1.33%
CS + DMARDs + MTX	2524	306.15 €	772,716.99 €	0.95%
CS + DMARDs + MTX + OTHER BIOLOGICS	229	6,631.73 €	1,518,665.63 €	1.87%
CS + DMARDs + OTHER BIOLOGICS	409	7,061.83 €	2,888,288.44 €	3.56%
DMARDs + MTX	1140	299.82 €	341,794.61 €	0.42%
DMARDs + MTX + OTHER BIOLOGICS	50	7,879.92 €	393,995.88 €	0.49%
DMARDs + OTHER BIOLOGICS	173	7,885.92 €	1,364,264.95 €	1.68%
	12363	2,138.17 €	26,434,153.87 €	32.55%
Total	35873	2,263.72 €	81,206,363.70 €	100.00

Note: CS = Corticosteroids, MTX = Methotrexate

## Discussion

Pharmaceutical cost, including cost of more expensive biologics, is considered one, if not the main, of the drivers of financial burden of RA on patients and health care systems. Understanding the real-world cost of pharmaceutical care for RA, on the basis of big data, is critical in evidence-based health services planning and resource allocation.

Our analysis revealed that 27.2% of patients on reimbursed treatment for RA are on a biologic containing treatment (monotherapy or combination, where the driver of the cost remains the biologic agent). This is higher than previously reported in the literature by Andrianakos et al. (14.05%) [3] and Sfikakis et al. (11.4%) [4] and may be attributed to the fact that patient share data is derived from the business intelligence database of EOPYY, which lists only reimbursed treatments. It is likely that there is a substantial number of patients that pay out of pocket for cheaper treatments, particularly for more moderate disease severity (such as methotrexate, corticosteroids and DMARDs) to avoid the cost of time for obtaining a prescription. Therefore, total number of patients with diagnosed RA on some treatment may be higher and, as a result, the percentage of patients on treatment with a biologic containing regimen smaller.

The mean annual cost per RA patient on reimbursed treatment for the study year was calculated at 2,263.72 €, which is slightly lower than the average spent per patient reported in 2008 [12] and the average EU cost [9]. This may be explained in part by the lower pharmaceutical prices in Greece–pharmaceuticals are priced at the average of the three lowest prices in EU28 and undergo a regular re-pricing exercise that leads to further price reductions.

At the time of our analysis, the Business Intelligence database did not include any biosimilars for the treatment of RA, as these were not yet available in the country. Biosimilars are highly comparable to their originator in terms of safety and efficacy and retailed at lower prices [13], thus contributing to cost savings. As biosimilar DMARDs are currently available in the market and their uptake increases, we can expect additional cost savings within the biologics category to be recorded on the database [14].

Our analysis excluded any indirect costs, which have been shown to account for a great, if not the greatest, part of total RA burden [15]. In Greece in particular, RA related indirect costs have been estimated at €2,492 per patient in a study conducted in 2008 [12]. Such a substantial burden, which is almost completely placed on patients and their family, is a critical input in global budget setting for the condition.

Furthermore, our analysis was limited to cost. The Business Intelligence database did not record any treatment outcomes or effectiveness. Therefore, we have been unable to evaluate the actual therapeutic benefits of access to treatment with biologics from an early age and on the basis of personalised treatment decisions (physician freedom of choice), which is expected to result in substantial cost savings in terms of inpatient care costs averted, as previously shown elsewhere [16–18]. This in itself is a finding of critical relevance to health care planning and management audiences: when designing and setting up national prescription monitoring databases, particularly for therapy areas with an increasing impact on healthcare budgets, it is imperative to be able to report on treatment outcomes, not just cost.

It is equally critical to evaluate how continued and uninterrupted access to such therapeutic options may help address or, on the contrary, exacerbate persistent inequalities in access to care for RA patients [19], particularly in the face of severe fiscal constraints and diminishing patient ability to pay out of pocket for health care [20].

## Conclusion

This is the first study to report on real life cost of pharmaceutical treatment for RA in Greece on the basis of big data. Our analysis confirms that the major driver of direct pharmaceutical expenditure is treatment with biologics, as a monotherapy or in combination with other therapeutic options, which appears a prevalent medical decision particularly for younger patients. The overall budget impact of access to such biologics from early on requires careful weighting against the respective therapeutic benefit to ensure continued and uninterrupted access and amelioration of any constraints, the latter being reported as prevalent amongst RA patients.

## References

[1] Scott DL, Wolfe F, Huizinga TWJ. Rheumatoid arthritis. Lancet. 2010;376(9746):1094–108. 10.1016/S0140-6736(10)60826-4 20870100

[2] McInnes IB, Schett G. The pathogenesis of rheumatoid arthritis. N Engl J Med. 2011;365(23):2205–19. 10.1056/NEJMra1004965 22150039

[3] Andrianakos A, Trontzas P, Christoyannis F, Kaskani E, Nikolia Z, Tavaniotou E, et al Prevalence and management of rheumatoid arthritis in the general population of Greece—the ESORDIG study. Rheumatology. 2006;45(12):1549–54. 10.1093/rheumatology/kel140 16690763

[4] Sfikakis P, Dafoulas G, Boumpas D, Drosos A, Kitas G, Liossis SN, et. al SAT0361 Large, Nation-Wide Data Analysis-Derived Estimated Prevalence of Rheumatoid Arthritis (RA), Systemic Lupus Erythematosus (SLE), and Systemic Sclerosis (SSC) in Caucasians: Insights from the Identification of Patients with Prescribed Pharmacological treatment among 7.742.629 Greek citizens. Ann Rheum Dis. 2015;74(Suppl 2):790.

[5] Borman P, Toy GG, Babaoğlu S, Bodur H, Ciliz D, Alli N. A comparative evaluation of quality of life and life satisfaction in patients with psoriatic and rheumatoid arthritis. Clin Rheumatol. 2007;26(3):330–4. 10.1007/s10067-006-0298-y 16622591

[6] Li X, Gignac MAM, Anis AH. The indirect costs of arthritis resulting from unemployment, reduced performance, and occupational changes while at work. Med Care. 2006;44(4):304–10. 10.1097/01.mlr.0000204257.25875.04 16565630

[7] Huscher D, Merkesdal S, Thiele K, Zeidler H, Schneider M, Zink A, et al Cost of illness in rheumatoid arthritis, ankylosing spondylitis, psoriatic arthritis and systemic lupus erythematosus in Germany. Ann Rheum Dis. 2006;65(9):1175–83. 10.1136/ard.2005.046367 16540552 PMC1798296

[8] Ozminkowski RJ, Burton WN, Goetzel RZ, Maclean R, Wang S. The impact of rheumatoid arthritis on medical expenditures, absenteeism, and short-term disability benefits. J Occup Environ Med. 2006;48(2):135–48. 10.1097/01.jom.0000194161.12923.52 16474262

[9] Kobelt G, Jönsson B. The burden of rheumatoid arthritis and access to treatment: outcome and cost-utility of treatments. Eur J Health Econ. 2008;8 Suppl 2:95–106. 18157559 10.1007/s10198-007-0091-0

[10] Souliotis K, Golna C, Kani C, Litsa P. Reducing patient copayment levels for topical and systemic treatments in plaque psoriasis as a case for evidence-based, sustainable pharmaceutical policy change in Greece. J Med Econ. 2016;19(11):1021–1026. 10.1080/13696998.2016.1192547 27207488

[11] Singh JA, Christensen R, Wells GA, Suarez-Almazor ME, Buchbinder R, Lopez-Olivo MA, et al A network meta-analysis of randomized controlled trials of biologics for rheumatoid arthritis: a Cochrane overview. CMAJ. 2009;181(11):787–96. 10.1503/cmaj.091391 19884297 PMC2780484

[12] Kobelt G, Kasteng F. Access to innovative treatments in rheumatoid arthritis in Europe. A report prepared for the European Federation of Pharmaceutical Industry Associations (EFPIA) Oct, 2009. http://www.comparatorreports.se/Access%20to%20RA%20Treatments%20October%202009.pdf

[13] Gulácsi L, Brodszky V, Baji P, Kim H, Kim SY, Cho YY, et al Biosimilars for the management of rheumatoid arthritis: economic considerations. Expert Rev Clin Immunol. 2015;11 Suppl 1:S43–52. 10.1586/1744666X.2015.1090313 26395836

[14] Smolen JS, Goncalves J, Quinn M, Benedetti F, & Lee JY. Era of biosimilars in rheumatology: reshaping the healthcare environment. RMD Open. 2019 5 21;5(1):e000900 10.1136/rmdopen-2019-000900 31245050 PMC6560670

[15] Benucci M, Rogai V, Atzeni F, Hammen V, Sarzti-Puttini P, Migliore A. Costs associated with rheumatoid arthritis in Italy: past, present, and future. Clinicoecon Outcomes Res. 2016;8:33–41. 10.2147/CEOR.S91006 26929654 PMC4754095

[16] Bonafede MMK, Gandra SR, Watson C, Princic N, Fox KM. Cost per treated patient for etanercept, adalimumab, and infliximab across adult indications: a claims analysis. Adv Ther. 2012;29(3):234–48. 10.1007/s12325-012-0007-y 22411424

[17] Schabert VF, Watson C, Joseph GJ, Iversen P, Burudpakdee C, Harrison DJ. Costs of tumor necrosis factor blockers per treated patient using real-world drug data in a managed care population. J Manag Care Pharm. 2013;19(8):621–30. 10.18553/jmcp.2013.19.8.621 24074008 PMC10438048

[18] Chevreul K, Haour G, Lucier S, Harvard S, Laroche M-L, Mariette X, et al Evolution of Direct Costs in the First Years of Rheumatoid Arthritis: Impact of Early versus Late Biologic Initiation—An Economic Analysis Based on the ESPOIR Cohort. Chopra A, editor. PLoS One. 2014;9(5):e97077 10.1371/journal.pone.0097077 24811196 PMC4014570

[19] Souliotis K, Papageorgiou M, Politi A, Ioakeimidis D, Sidiropoulos P. Barriers to accessing biologic treatment for rheumatoid arthritis in Greece: the unseen impact of the fiscal crisis—the Health Outcomes Patient Environment (HOPE) study. Rheumatol Int. 2014;34(1):25–33. 10.1007/s00296-013-2866-1 24057144

[20] Kanavos P, and Souiotis K, (2017). Reforming the Healthcare in Greece: Balancing Fiscal Adjustment with Healthcare Needs, pp. 359–401, in: Meghir C, Pissarides C, Vayanos D, and Vettas N. (eds.): Beyond Austerity: reforming the Greek Economy, MIT Press, Cambridge, Massachusetts.

